# Effect of Pupil Size on Optical Quality Parameters in Astigmatic Eyes Using a Double-Pass Instrument

**DOI:** 10.1155/2013/124327

**Published:** 2013-06-23

**Authors:** Hidenaga Kobashi, Kazutaka Kamiya, Kyohei Yanome, Akihito Igarashi, Kimiya Shimizu

**Affiliations:** Department of Ophthalmology, University of Kitasato School of Medicine, 1-15-1 Kitasato, Minami, Sagamihara, Kanagawa 252-0374, Japan

## Abstract

*Purpose*. To objectively determine the effects of pupil size on optical quality parameters in astigmatic eyes using a double-pass instrument. *Methods*. We examined twenty-two eyes of 22 healthy volunteers (mean age ± standard deviation, 27.1 ± 2.8 years) who had no ophthalmic diseases other than refractive errors (manifest cylinder ≤0.25 diopters (D)). After we fully corrected cycloplegic refraction, we created with-the-rule astigma tism of 1, 2, and 3 diopters in these eyes and then quantitatively assessed the modulation transfer function (MTF) cutoff frequency and the Strehl2D ratio with 2-, 4-, and 6-mm pupil sizes using the Optical Quality Analysis System. *Results*. The MTF cutoff frequency and the Strehl2D ratio decreased significantly as the amount of astigmatism increased at each pupil size (*P* < 0.001 for 2, 4, and 6 mm, analysis of variance). They also decreased significantly with an increase in pupil size at each diopter of astigmatism (*P* < 0.001 for 0, 1, 2, and 3 D). Multiple comparisons demonstrated a significant difference between measurements made for a 2-mm pupil and for a 6-mm pupil at each diopter of astigmatism (*P* < 0.001 for 0, 1, 2, and 3 D, Dunnett test) and those made for a 4-mm pupil and for a 6-mm pupil at each diopter of astigmatism (*P* < 0.001 for 0 D, *P* < 0.05 1, 2, and 3 D). *Conclusions*. Eyes with larger pupils had lower optical quality even when they were astigmatic. It may be necessary to correct the preexisting astigmatism to acquire excellent visual performance, especially in astigmatic eyes with larger pupils.

## 1. Introduction

Although modern refractive and cataract surgery techniques allow rapid visual recovery, preexisting corneal astigmatism remains a common change to achieve excellent uncorrected visual acuity. Moreover, it has been estimated that approximately 30%, 8%, and 3% of patients have more than 1, 2, and 3 diopters (D), respectively, of preexisting cylindrical refractive errors [1–3]. Several surgical options for the correction of astigmatism have been advocated, such as laser in situ keratomileusis, photorefractive keratectomy, astigmatic keratotomy using limbal or corneal relaxing incisions, and a toric phakic or pseudophakic intraocular lens implantation. At present, we merely consider the amount of astigmatism as well as the axis of astigmatism for these surgical approaches.

The optical quality analysis system (OQAS, Visiometrics, Terrassa, Spain), which is designed on the basis of the asymmetric pattern of the double-pass technique, has been shown to be a useful tool for comprehensively evaluating the optical quality of the eye [4–7]. Moreover, it has been demonstrated that this device has excellent repeatability of measurement [6, 7]. We recently showed that pupil size may play an essential role in visual acuity in eyes having astigmatism [8]. However, to our knowledge, the effects of pupil size on optical quality parameters, such as the modulation transfer function (MTF) cutoff frequency or the Strehl ratio, have not so far been objectively assessed in such eyes. The purpose of this study is to prospectively evaluate the effects of pupil size on optical quality parameters in astigmatic eyes using the double-pass instrument.

## 2. Materials and Methods

### 2.1. Study Subjects

 Twenty-two eyes of 22 healthy volunteers (12 female, 10 male; mean age ± standard deviation (SD), 27.1 ± 2.8 years) were enrolled in this prospective study. The inclusion criteria for this study were as follows: best spectacle-corrected visual acuity of 20/20 or better, manifest refractive cylinder of 0.25 diopters (D) or less, manifest spherical equivalent of -4 D or less, and no history of any ophthalmic disease, or ocular surgery. Eyes with keratoconus were excluded from the study by using the keratoconus screening test of Placido disk videokeratography (TMS-2, Tomey, Nagoya, Japan). Ocular higher-order aberrations (HOAs) for a 4-mm pupil were determined with Hartmann-Shack aberrometry (KR-9000, Topcon, Tokyo, Japan). The sample sizes in our study offered 85% statistical power at the 5% level to detect a 10-cycles/degree difference in MTF cut-off frequencies between the two groups, when the SD of the mean difference was 15.0 cycles/degree. They also offered 85% statistical power at the 5% level to detect a 0.04-difference in the Strehl ratio between the two groups, when the SD of the mean difference was 0.06. The study was approved by the Institutional Review Board at Kitasato University, School of Medicine. Written informed consent was obtained from all patients after explanation of the nature and possible consequences of the study. 

### 2.2. Experimental Procedure

Manifest and noncycloplegic refractions were examined with the autorefractor (ARK-700A; Nidek, Gamagori, Japan). Cycloplegia was achieved with three drops of 1% cyclopentolate hydrochloride (Cyplegin; Santen, Osaka, Japan), spaced 5 minutes apart. The autorefractor was also performed at least 30 minutes after the third administration of cyclopentolate hydrochloride and only if the pupillary light reflex was absent. One eye of each subject was chosen randomly for the measurement. Three consecutive sets of measurements with the device were performed in all subjects by a single experienced examiner (H.K.) and were then averaged for statistical analysis. The ARK-700A autorefractor has been reported to have excellent repeatability of the measurements [9]. The manifest refractive error of the subjects was fully corrected during these measurements; the spherical error was automatically corrected by the double-pass system, and the cylindrical error was corrected with an external lens, because the uncorrected refractive error directly affects the optical outcome of the system. To determine the optical quality of different pupil sizes in astigmatic eyes, we varied the artificial pupil size from 2 to 6 mm. If a minor residual astigmatism exists, the subject looks for focusing on the circle of least confusion. Without changing this focal condition, we varied the cylindrical lens power between 1 D and 3 D undercorrection of the astigmatism, at 1-D intervals. In these cases the retinal image of the point source is a Sturm focal. With regard to axis orientation in astigmatic eyes, we assessed optical quality parameters in eyes with with-the-rule astigmatism.

### 2.3. Optical Quality Measurement

We measured the optical quality parameters of the eye, such as the MTF cutoff frequency and the Strehl2D ratio, in these eyes using the OQAS. Near-infrared light consisting of a laser diode (wavelength, 780 nm) is used because it is more comfortable for the subject and provides retinal image quality estimates that are comparable to those obtained with visible light. The natural pupil diameter was provided by this device from an image of an additional video camera that allowed pupil alignment. We confirmed that the natural pupil diameter was more than 6.0 mm after cycloplegia. The size of the artificial pupil is controlled by means of a diaphragm wheel located inside the double-pass system. The room illumination was kept low (approximately 25 lux) during testing. 

From the retinal image of each analyzed eye, the monochromatic MTF was computed. The MTF represents the loss of contrast as a function of the spatial frequency. A two-dimensional radially averaged profile of the MTF is used to describe the complete eye's optical quality in the double-pass instrument. To simplify the data and facilitate the clinical comparison of retinal image quality between subjects, the system provides several parameters that are related to the MTF: the MTF cutoff frequency and the Strehl2D ratio.

The value considered is the cutoff point of the MTF curve on the *x*-axis; the results are given in cycles per degree, representing the highest spatial frequency at lower contrast. The MTF cutoff in the double-pass system is the frequency at which the MTF reaches a value of 0.01. Because the point spread function (PSF) images recorded by the double-pass instrument can be affected by high-frequency noise, which is inherent in the use of cameras, the frequency for very small MTF values may become unstable, potentially leading to artifacts. To avoid this problem, the device uses an MTF threshold value of 0.01, which corresponds to 1% contrast. Thus, the MTF cutoff frequency in this paper refers to the frequency up to which the eye can focus an object on the retina with a significant 1% contrast. 

In the visual optics field, the Strehl ratio is often computed in the frequency domain as the ratio between the volume under the MTF curve of the measured eye and that of the aberration-free eye [10]. This provides overall information on the eye's optical quality. The double-pass system computes the Strehl ratio in two dimensions (Strehl2D ratio) as the ratio between the area under the MTF curve of the measured eye and that of the aberration-free eye. This computation has a lower cost in time, which makes it more suitable for clinical practice. A Strehl ratio of 1 is related to a perfect optical system that is only limited by diffraction. The poor-quality data (e.g., blinking artifacts or poor detections of PSF images by the software) were manually excluded.

In addition, to assess the repeatability of the measurements for confirming the applicability of the data, the measurements with this device were made in 22 eyes with a 4 mm pupil at 2 D of astigmatism at the same time of day on two days. We evaluated the repeatability of the two measurements as described previously using Bland-Altman plots [11].

### 2.4. Statistical Analysis

All statistical analyses were performed using SPSS (SPSS Inc, Chicago, IL, USA). One-way repeated-measures analysis of variance (ANOVA), followed by the Dunnett post hoc test for multiple comparisons, was used to compare the differences between groups with different pupil sizes. The results are expressed as mean ± standard deviation, and a *P* value of <0.05 was considered statistically significant. 

## 3. Results

Table 1 shows the demographic data of the study population. We found no significant differences between manifest, noncycloplegic, and cycloplegic refractions (*P* = 0.99 for spherical equivalent refraction, *P* = 0.54 for cylindrical refraction, ANOVA).

**Figure 1:**
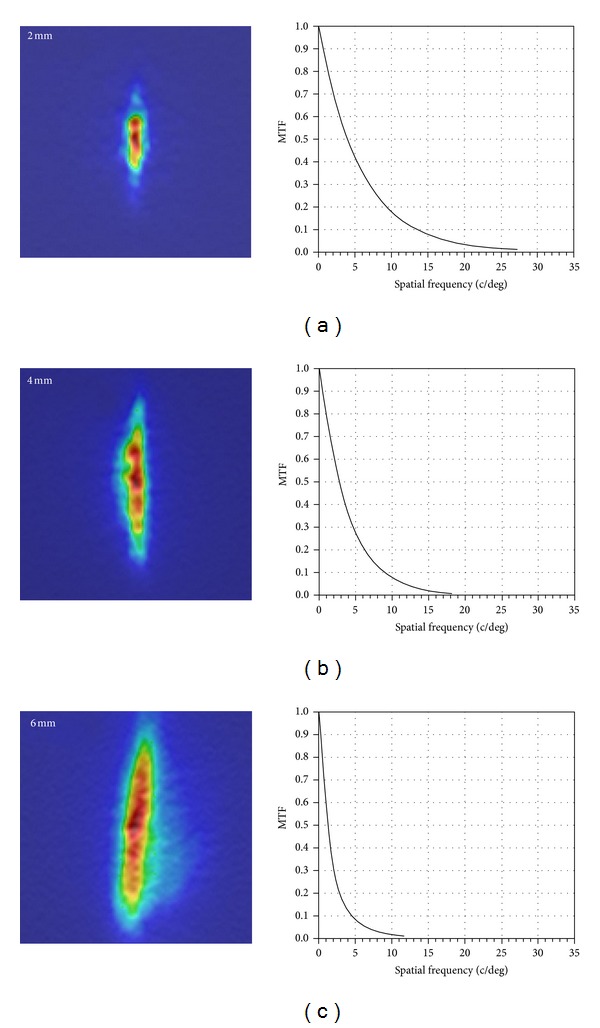
Point spread function (PSF) (left) and modulation transfer function (MTF) (right) images of 3 diopters of astigmatism obtained by the Optical Quality Analysis System: (a) 2-mm pupil, (b) 4-mm pupil, and (c) 6-mm pupil.

Figure 1 shows representative examples of the double-pass images in eyes with 3 D of astigmatism at the different pupil sizes (2, 4, and 6 mm). Our results provided by the instrument were analyzed in the low-power principal meridian which showed maximum elongation of the double-pass image.

**Figure 2:**
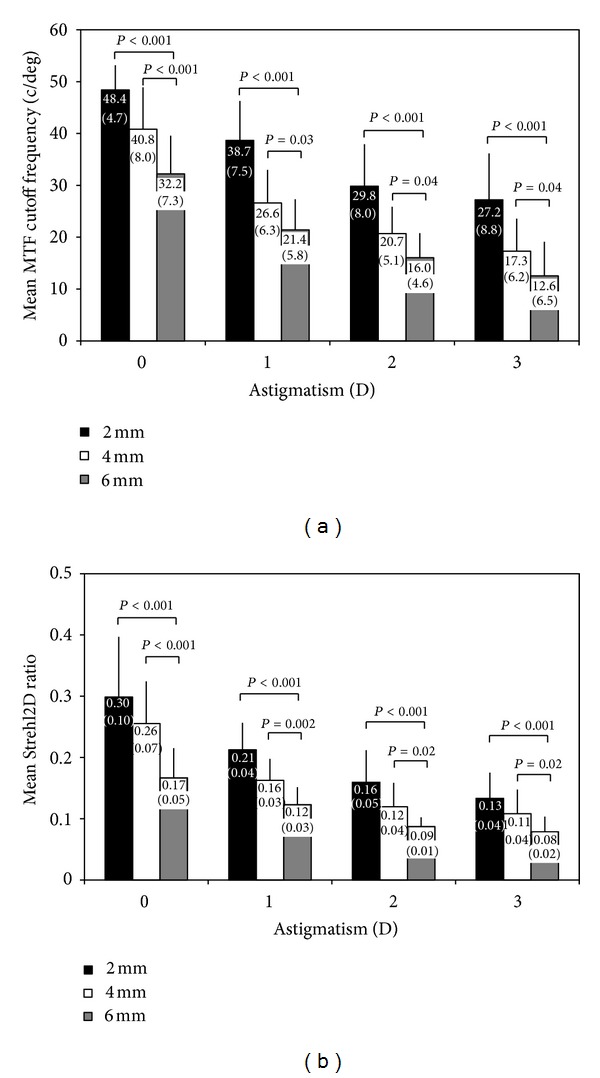
Optical quality parameters with 2-, 4-, and 6-mm pupil sizes at each diopter of astigmatism. (a) Mean modulation transfer function (MTF) cutoff frequency. (b) Mean Strehl2D ratio. Each parenthesis means values of the standard deviations. Repeated-measures analysis of variance, followed by the Dunnett post hoc test for multiple comparisons, was used to compare the differences between groups with different pupil sizes: c/deg = cycles per degree, D = diopter.

The MTF cutoff frequency decreased significantly as the amount of astigmatism increased (*P* < 0.001 for 2, 4, and 6 mm, ANOVA). It was also significantly affected by pupil size at each diopter of astigmatism (*P* < 0.001 for 0, 1, 2, and 3 D, ANOVA). Multiple comparisons demonstrated a significant difference between measurements made for a 2 mm pupil and for a 6 mm pupil at each diopter of astigmatism (*P* < 0.001 for 0, 1, 2, and 3 D, Dunnett test) and those made for a 4 mm pupil and for a 6 mm pupil at each diopter of astigmatism (*P* < 0.001 for 0 D, *P* = 0.03 for 1 D, *P* = 0.04 for 2 and 3 D, Figure 2).

The Strehl2D ratio also decreased significantly as the amount of astigmatism increased (*P* < 0.001 for 2, 4, and 6 mm, ANOVA). It was also significantly affected by pupil size at each diopter of astigmatism (*P* < 0.001 for 0, 1, 2, and 3 D, ANOVA). Multiple comparisons demonstrated a significant difference between measurements made for a 2 mm pupil and for a 6 mm pupil at each diopter of astigmatism (*P* < 0.001 for 0, 1, 2, and 3 D, Dunnett test), and those made for a 4 mm pupil and for a 6 mm pupil at each diopter of astigmatism (*P* < 0.001 for 0 D, *P* = 0.002 for 1 D, *P* = 0.02 for 2 and 3 D, Figure 2). All optical quality parameters were summarized in Table 2.

**Figure 3:**
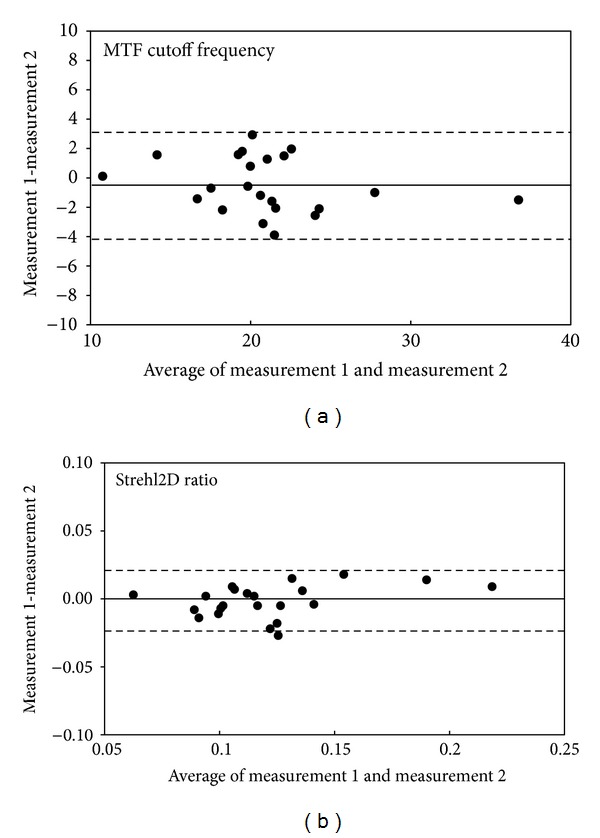
Bland-Altman plots represent the difference between two measurements divided by the mean of these measurements. (a) Modulation transfer function (MTF) cutoff frequency (b) Strehl2D ratio. The solid lines represent mean differences between 2 measurements of MTF cutoff frequency and Strehl2D ratio; dotted lines are the upper and lower borders of the 95% limit of agreement (mean difference ± 1.96 multiplied by standard deviation of the mean difference).

Bland-Altman plots indicate that the mean difference between two measurements with this device (± 95% limits of agreement; LoA) was −0.05 ± 1.89 cycles/degree (− 4.18 to 3.24 cycles/degree) for MTF cutoff frequency and 0.00 ± 0.01 (− 0.03 to 0.02) for Strehl2D ratio (Figure 3).

## 4. Discussion

The results of our study have revealed that both the MTF cutoff frequency and the Strehl2D ratio were better in eyes with less astigmatism at each pupil size. It is quite reasonable that this was in line with previous studies in which eyes with a greater astigmatism had lower optical quality [12, 13]. Our results have also shown that astigmatic eyes with larger pupil sizes had lower optical quality than those with smaller pupil sizes, which was consistent with their previous findings in nonastigmatic eyes [14–16], indicating that pupil size plays an essential role in visual performance in astigmatic eyes as well as in nonastigmatic eyes. Since the level of HOAs appears to be low and almost equivalent in all eyes, as shown in Table 1, we assume that HOAs did not significantly influence the visual outcomes in this study. We believe that our findings are informative to most surgeons because they do not evaluate pupil size for the surgical correction of astigmatism in a clinical setting. As far as we can ascertain, this is the first study to objectively assess the effect of pupil size on detailed optical quality parameters, such as the MTF cutoff frequency or the Strehl2D ratio, in astigmatic eyes. Coupled with our previous [8] and current findings, it may be necessary to correct the preexisting astigmatism to acquire excellent visual outcomes, especially in astigmatic eyes with larger pupils, from the subjective and objective viewpoints.

It is of clinical importance to assess the repeatability of the measurements with this instrument in order to confirm the applicability of the data. It has been demonstrated that the device has good repeatability [6, 7], and that the realignment of the eyes does not impose any additional variation on the measurements [6, 7]. As shown in Figure 3, we confirmed the good repeatability of the measurements in the current study, as evidenced by the narrow 95% LoA. Hence, we believe that this device offers reasonable repeatability in the clinical evaluation of the optical quality of the eye.

There are at least two limitations to this study. First, we used the artificial pupils, which were not at all influenced by these factors, at the spectacle plane, with the OQAS under cycloplegia in the present study. It is known that pupil size can be influenced not only by patient background, for example, by age, manifest refraction, and the accommodative state of the eye, and by various sensory and emotional conditions, but also by measurement conditions affecting the level of retinal illuminance. A further study is needed in order to clarify the exact role of pupil size on visual performance under natural viewing conditions. Second, we objectively assessed optical quality parameters only in eyes with with-the-rule astigmatism. However, these values should be theoretically constant at each type of astigmatism even if the axis orientation was changed, since the PSF image itself was rotated symmetrically with respect to a central point. Moreover, in our preliminary data, we also obtained almost similar results not only in eyes with against-the-rule astigmatism, but also in those with oblique astigmatism (data not shown). Therefore, we believe that the axis orientation does not significantly affect these objective parameters in the current study.

## 5. Conclusions

In summary, our results demonstrated that astigmatic eyes with larger pupil sizes had lower objective visual performance, such as a lower MTF cutoff frequency and a lower Strehl2D ratio. It is suggested that not only subjective but also objective visual performance was influenced by pupil size in astigmatic eyes. We believe that these findings, although simple, are clinically meaningful since most surgeons consider merely the amount of astigmatism and the astigmatic axis for the surgical correction of astigmatism in a clinical setting.

## Figures and Tables

**Table 1:** Demographics of the study population.

	Mean ± SD (range)
Age (years)	27.1 ± 2.8 (23 to 33)
Gender (% female)	54.5%

Spherical equivalent refraction	

Manifest (D)	−1.02 ± 1.53 (−3.25 to 0.75)
Noncycloplegia (D)*	−0.98 ± 1.38 (−3.75 to 0.50)
Cycloplegia (D)*	−0.96 ± 1.35 (−3.75 to 0.50)

Cylindrical refraction	

Manifest (D)	−0.19 ± 0.21 (−0.25 to 0.00)
Noncycloplegia (D)*	−0.14 ± 0.19 (−0.25 to 0.00)
Cycloplegia (D)*	−0.13 ± 0.13 (−0.25 to 0.00)
Ocular HOAs (*μ*m, for a 4-mm pupil)	0.11 ± 0.03 (0.04 to 0.17)

D: diopter, SD: standard deviation, HOAs: higher-order aberrations, *Measured using an autorefractor.

**Table 2:** Optical quality parameters of different pupil sizes in astigmatic eyes.

Parameters	Astigmatism	Artificial pupil size		
		2 mm	4 mm	6 mm
MTF cutoff frequency (c/deg)	0 D	48.4 ± 4.7	40.8 ± 8.0	32.2 ± 7.3
	1 D	38.7 ± 7.5	26.6 ± 6.3	21.4 ± 5.8
	2 D	29.8 ± 8.0	20.7 ± 5.1	16.0 ± 4.6
	3 D	27.2 ± 8.8	17.3 ± 6.2	12.6 ± 6.5

Strehl2D ratio	0 D	0.30 ± 0.10	0.26 ± 0.07	0.17 ± 0.05
	1 D	0.21 ± 0.04	0.16 ± 0.03	0.12 ± 0.03
	2 D	0.16 ± 0.05	0.12 ± 0.04	0.09 ± 0.01
	3 D	0.13 ± 0.04	0.11 ± 0.04	0.08 ± 0.02

ANOVA: analysis of variance, MTF: modulation transfer function, D: diopters.

## References

[1] Read SA, Collins MJ, Carney LG (2007). A review of astigmatism and its possible genesis: invited review. *Clinical and Experimental Optometry*.

[2] Ferrer-Blasco T, Montés-Micó R, Peixoto-de-Matos SC, González-Méijome JM, Cerviño A (2009). Prevalence of corneal astigmatism before cataract surgery. *Journal of Cataract & Refractive Surgery*.

[3] Hoffmann PC, Hütz WW (2010). Analysis of biometry and prevalence data for corneal astigmatism in 23 239 eyes. *Journal of Cataract and Refractive Surgery*.

[4] Güell JL, Pujol J, Arjona M, Diaz-Douton F, Artal P (2004). Optical Quality Analysis System: instrument for objective clinical evaluation of ocular optical quality. *Journal of Cataract and Refractive Surgery*.

[5] Vilaseca M, Padilla A, Pujol J, Ondategui JC, Artal P, Güell JL (2009). Optical quality one month after Verisyse and Veriflex phakic IOL implantation and Zeiss MEL 80 LASIK for myopia from 5.00 to 16.50 diopters. *Journal of Refractive Surgery*.

[6] Saad A, Saab M, Gatinel D (2010). Repeatability of measurements with a double-pass system. *Journal of Cataract and Refractive Surgery*.

[7] Vilaseca M, Peris E, Pujol J, Borras R, Arjona M (2010). Intra- and intersession repeatability of a double-pass instrument. *Optometry and Vision Science*.

[8] Kamiya K, Kobashi H, Shimizu K, Kawamorita T, Uozato H (2012). Effect of pupil size on uncorrected visual acuity in astigmatic eyes. *British Journal of Ophthalmology*.

[9] Allen PM, Radhakrishnan H, O’Leary DJ (2003). Repeatability and validity of the PowerRefractor and the Nidek AR600-A in an adult population with healthy eyes. *Optometry and Vision Science*.

[10] Navarro R, Artal P, Williams DR (1993). Modulation transfer of the human eye as a function of retinal eccentricity. *Journal of the Optical Society of America A*.

[11] Bland JM, Altman DG (1986). Statistical methods for assessing agreement between two methods of clinical measurement. *The Lancet*.

[12] Mitchell DE, Wilkinson F (1974). The effect of early astigmatism on the visual resolution of gratings. *Journal of Physiology*.

[13] Pujol J, Arjona M, Arasa J, Badia V (1998). Influence of amount and changes in axis of astigmatism on retinal image quality. *Journal of the Optical Society of America A*.

[14] Atchison DA, Smith G, Efron N (1979). The effect of pupil size on visual acuity in uncorrected and corrected myopia. *American Journal of Optometry and Physiological Optics*.

[15] Artal P, Navarro R (1994). Monochromatic modulation transfer function of the human eye for different pupil diameters: an analytical expression. *Journal of the Optical Society of America A*.

[16] Artigas JM, Menezo JL, Peris C, Felipe A, Díaz-Llopis M (2007). Image quality with multifocal intraocular lenses and the effect of pupil size. Comparison of refractive and hybrid refractive-diffractive designs. *Journal of Cataract and Refractive Surgery*.

